# Toward a general theoretical framework for judgment and decision-making

**DOI:** 10.3389/fpsyg.2015.00159

**Published:** 2015-02-17

**Authors:** Davide Marchiori, Itzhak Aharon

**Affiliations:** ^1^Strategic Organization Design Unit, Department of Marketing and Management, University of Southern DenmarkOdense, Denmark; ^2^Lauder School of Government, Diplomacy and Strategy, School of Psychology, Interdisciplinary Center HerzliyaHerzliya, Israel

**Keywords:** behavioral economics, decisions from experience, neuroscience, psychology, judgment and decision-making

Over the past 30 years, behavioral and experimental economists and psychologists have made great strides in identifying phenomena that cannot be explained by the classical model of rational choice—anomalies in the discounting of future wealth, present bias, loss aversion, the endowment effect, and aversion to ambiguity, for example. In response to these findings, there has been an enormous amount of research by behavioral scientists aimed at modeling and understanding the nature of these biases^1^. However, these models, typically assuming situation-specific psychological processes, have shed limited light on the conditions for and boundaries of the different biases, substantially neglecting their relative importance and joint effect. Much less attention has been paid to the investigation of the links between different biases. As a consequence of this approach, it is not always clear which model should be used to predict behavior in a new setting, and maybe a more general theory is needed. We believe that the field of neuroeconomics, which has experienced a rapid growth over the past decade, can play an important role in bridging these gaps, contributing to the building of a general theoretical framework for judgment and decision-making behaviors.

## Apparently inconsistent biases

One of the main insights from decision-making studies is that people tend to overweight small probability events in risky one-shot decisions (Kahneman and Tversky, 1979). This tendency can explain why, for example, people buy lottery tickets and insurance. However, one might wonder, for instance, why in most Western Countries driving insurance is compulsory (how many drivers would spontaneously ensure?); Why the enforcement of safety rules at the workplace and of safe medical procedures have become social issues of primary importance, causing massive public and private investments (Erev et al., 2010); or why only a small share of people actually participate in lotto games on a regular basis (Pérez and Humphreys, 2011). Answering such questions, recent experimental studies have shown that in repeated decisions with feedback, people tend to *underweight* small probability events, and behave as if “it won't happen to me” (Barron and Erev, 2003; Hertwig and Erev, 2009)^2^.

We could simply conclude that people tend to overweight rare events in one-shot decisions from description, and underweight rare events in experience-based decisions. Unfortunately, this assertion cannot predict behavior in a situation in which decision makers are provided with both the description of the incentive structure and feedback about their own choices (see Lejarraga and Gonzalez, 2011). To better illustrate this problem, consider the situation in which people can choose whether to insure against rare devastating natural events, whose occurrence rate and effect are known (see Marchiori et al., 2015): Will people be willing to buy insurance at, for example, the price that equals the expected cost from the risk? If models of one-shot decisions from description seem to give a positive answer to this question^3^, models of decisions from experience suggest the opposite^4^. Which theory should inform an insurance pricing policy?

Situation-specific psychological processes have also been proposed to explain other aspects of decision-making, such as the tendency to explore new alternatives. Empirical evidence suggests that people appear to insufficiently explore new alternatives in some situations (the most popular example is the preference for the status quo, as shown by Samuelson and Zeckhauser, 1988), whereas they exhibit the opposite tendency in others (e.g., unsafe sexual behavior and use of illicit drugs; see, for example, Bechara, 2005). Again, one could be tempted to explain these apparently contradicting phenomena by asserting that in some settings people tend to explore insufficiently, whereas in others they exhibit the opposite bias.

This different-biases-different-explanations approach has not spared the judgment field. As a result, two important streams of judgment research have led to apparently contradicting conclusions: Whereas revision-of-opinion studies hold that judgment is affected by conservatism (e.g., Phillips and Edwards, 1966)^5^, calibration studies demonstrate that judgment is affected by the opposite bias—overconfidence (e.g., Fischhoff et al., 1977)^6^. Once again, one might explain this apparent contradiction by assuming that in some settings judgment is conservative, and overconfident in others.

Are the pairs of judgment and decision-making biases mentioned in the previous paragraphs really inconsistent, justifying the development of specific, *post-hoc* theories? Or is it possible to formulate a more general theory that provides the (sufficient) conditions for the different biases, as well as their relative importance and joint effect? The field of behavioral sciences lacks of such a theory (see Camerer and Loewenstein, 2004; Erev and Greiner, 2015), but some important steps in this direction have recently been made. For example, the contribution by Erev et al.'s (1994) addresses this methodological issue within the judgment field. This paper shows that conservatism and overconfidence can coexist, and proposes a common theoretical explanation for the two biases—the assertion that human judgment is affected by random errors. Along this line, Dougherty et al.'s (1999) MINERVA-DM memory model accounts not only for the simultaneous coexistence of conservatism and overconfidence (besides other biases affecting judgment), but also for the effect of experience on the relative importance of these two biases.

In the decision making domain, a recent line of research has been trying to shed light on the *boundaries* of some well-known phenomena documented in previous literature. This is the case of the recent discussions about the robustness and generality of loss aversion provided by, for example, Ert and Erev (2008, 2013) and Yechiam and Hochman (2014)^7^; the conditions for underweighting of rare events (e.g., Rakow et al., 2008); or the construct of risk taking by Yechiam and Telpaz (2011, 2013).

In another recent line of decision-making studies, researchers have been trying to clarify the (sufficient) conditions under which different biases are likely to occur. Studies in this domain abandon the (subjective) value function metaphor, and demonstrate the value of the assumption that choice behavior is driven by past experiences in similar situation (see Erev and Haruvy, 2014)^8^. This research trend includes, for example, the contributions by Erev et al. (2015), which analyzes choice behavior in negative-sum betting games; Teodorescu and Erev (2014), which highlights the conditions that lead to under- and over-exploration in the context of multi-alternative repeated decision tasks with no description and limited feedback; and by Zion et al. (2010), which analyzes financial choice behavior. Remarkably, most of the results reported in these studies cannot be accounted for by mainstream behavioral models (e.g., expected payoff maximization, risk aversion, loss aversion, and the possibility effect).

The contribution by Marchiori et al. (2015) pushes further this experiential line of decision-making research, extending Erev et al.'s (1994) conceptual analysis and Dougherty et al.'s (1999) MINERVA-DM memory model to the decision-making domain. Marchiori et al.'s demonstrate the predictive value of models assuming reliance on small samples of past experience and overgeneralization (intended as the tendency to confound instances of previously encountered tasks that are perceived as similar to the decision problem at hand). Marchiori et al.'s model provides sufficient conditions for two pairs of apparently contradicting phenomena documented in the judgment and decision-making field: Over- and under-weighting of rare events, and over- and under-estimation of low probabilities.

## A change in perspective

To cope with behaviors that cannot be accounted for by the rational framework, behavioral economists and psychologists have developed insightful theories and models of judgment and decision-making. However, the dominant methodology has consisted in building models that account for behavioral anomalies in specific settings. As a result, this methodological approach has contributed to the fragmentation of the field of behavioral economics, and produced models whose predictive power is often limited. These problems prompt the question of what might be the candidate theoretical framework for the development of a more general theory of choice behavior, alternative to the rationality paradigm.

The recent developments in the decision making field mentioned earlier show that the approach emphasizing the primary role of past experience as driver of choice behavior can overcome the methodological issues that have since long accompanied the field of behavioral economics and—more in general—that of behavioral sciences. This is possible as this perspective has been shown to provide a coherent and powerful framework for judgment and decision making modeling (see, for example, Juslin et al., 2007; Nevo and Erev, 2012).

However, that individuals make decisions based on small samples of past experiences in similar situations has to be interpreted as an “as if” explanation, thus prompting the question of to what extent does this abstraction correspond to the processes that *actually* occur in the human brain (cf. Yechiam and Aharon, 2011). Results from neuroscience have already been proven to be a valuable resource for the improvement of economic models of learning and choice behavior by highlighting, for example, the physiology of emotions that affect decision making^9^, and by suggesting new and more physiologically plausible modeling techniques (Marchiori and Warglien, 2008, 2011).

In addition, research in neuroscience could be very helpful in shedding light on the mechanisms underlying decision-making at the individual level. Specifically, a question of central importance for the experiential research line of decision-making is about how individuals assess similarity between present and past experiences. Understanding this process by means of the traditional tools of empirical behavioral investigation appears to be a very difficult task: Compared to the number of observations typically available in a behavioral study, individual data appear to be affected by too much noise and the number of free parameters of a comprehensive model of individual behavior would be too large. In this respect, we suggest that the contribution of neuroscience will be crucial in pinpointing and clarifying the physical processes according to which past experiences are reinforced and mapped onto newly encountered judgment and decision making problems. Particularly relevant to this issue is the recent neuro-research on how past experience is used to imagine future happenings and scenarios and how analogy links between different situations are established at the brain level (see, for example, Buckner and Carroll, 2007; Schacter et al., 2007; Boyer, 2008; Bar, 2009).

The emerging fields of genoeconomics, geno-neuroeconomics, and evolutionary neuro-social science might help understanding to what extent individual behavior is learnt and to what extent it is affected by genetics, and shed light on the important issue of individual heterogeneity in choice behavior, which has not yet been exhaustively explained by behavioral studies. Along these directions, recent studies in these emerging fields have already tried to investigate how genetics affects individual heterogeneity in choice behavior (Parasuraman and Jiang, 2012), how risk taking in investment portfolios is linked to genetic variability (Cesarini et al., 2010), the heritability of aspects of consumer judgment and choice behavior (Simonson and Sela, 2011), or how natural selection has shaped modern human behavior (Lieberman et al., 2003).

### Conflict of interest statement

The authors declare that the research was conducted in the absence of any commercial or financial relationships that could be construed as a potential conflict of interest.

## Footnotes

^1^Including the line of research that Gigerenzer and Selten (2002) refer to as “Subjective Expected Utility (SEU) repair program.” The main shortcomings of this approach have been recently pointed out by Erev and Roth (2014).

^2^Underweighting of rare events can be nicely captured by the assertion that decision makers tend to rely on *small* samples of past experiences in similar situations (Hau et al., 2008; Nevo and Erev, 2012).

^3^This is what Prospect Theory predicts, assuming a convex value function for losses (see Kahneman and Tversky, 1979).

^4^As predicted by models that assume best reply to the information conveyed by small samples of past experiences. According to this abstraction, decision-makers' preferences are biased toward the option whose outcome is reinforcing most of the times—in the current example, the risky option of not to insure.

^5^In the typical experiment, participants are asked the probability of an event after having collected relevant data. In this setting, judgment tends to be *less* extreme than the corresponding objective probabilities.

^6^In the typical experiment, participants are asked to express their confidence that statements or answers to general questions are accurate. In this setting, judgment is observed to be *more* extreme than the average accuracy rate.

^7^The contributions by Yechiam, Hochman, and Telpaz also analyze a cognitively-grounded explanation for the effect of losses on choice behavior, alternative to loss aversion. This alternative explanation holds that losses increase subjects' on-task attention, which in its turn increases subjects' sensitivity to the reinforcement structure of the decision task at hand. This attention-based account is supported by data from behavioral, brain, and pupilometry studies.

^8^This idea is not new in Psychology. For example, Skinner used it to explain why people “are more reluctant to buy a second ticket to the theater after losing a first one, than after losing the money they had set aside for that purpose” (Skinner, 1985:297). Instead of a reflection of different categorizations (as proposed by Kahneman and Tversky, 1984), Skinner suggests that in the two described situations people recall different kinds of past experiences (which he referred to as *contingencies of reinforcement*): On the one hand, experiences involving protesting for something we have already done and we refuse to repeat, and, on the other, experiences involving (prompt) replacement of spoiled or lost objects.

^9^Brain studies on the role of regret on decision making are perhaps the most popular example (e.g., Camille et al., 2004; Coricelli et al., 2005).
